# Supplementary material for: The role of connectivity on COVID-19 preventive approaches
Source: medRxiv. 2021 May 25:2021.03.11.21253348. Originally published 2021 Mar 12. Preprint. [Version 3] doi: 10.1101/2021.03.11.21253348 (PMC7987035; doi:10.1101/2021.03.11.21253348)
Supplement: 1 [file NIHPP2021.03.11.21253348V3-supplement-1.pdf]

medRxiv preprint doi: https://doi.org/10.1101/2021.03.11.21253348; this version posted May 25, 2021. The copyright holder for this preprint (which was not certified by peer review) is the author/funder, who has granted medRxiv a license to display the preprint in perpetuity.
It is made available under a CC-BY 4.0 International license .

**Supplementary Figures**

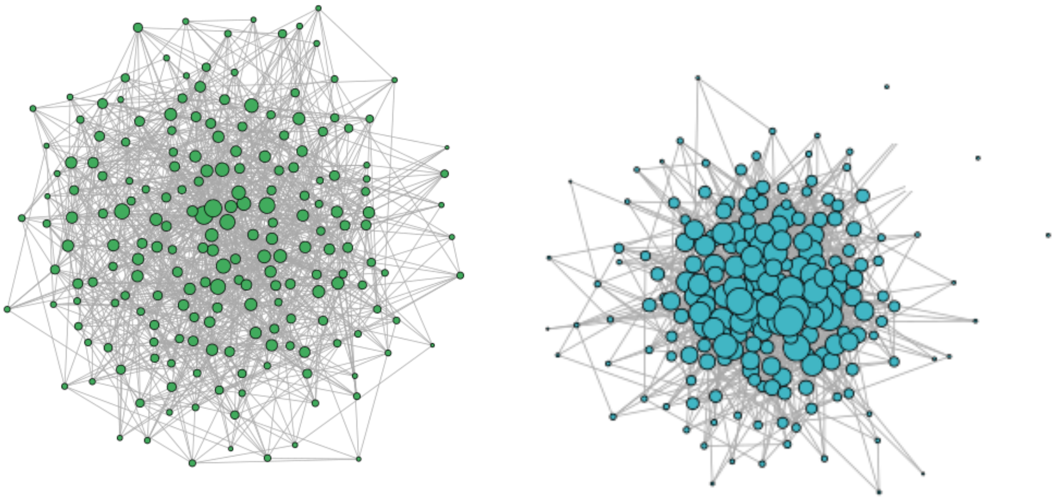

**S1 Fig.** An Erdős-Rényi graph (left hand side) and a power-law degrees graph (right hand side). For an easy visualisation, the parameters where set to N = 200, e = 12, $\lambda$=3.

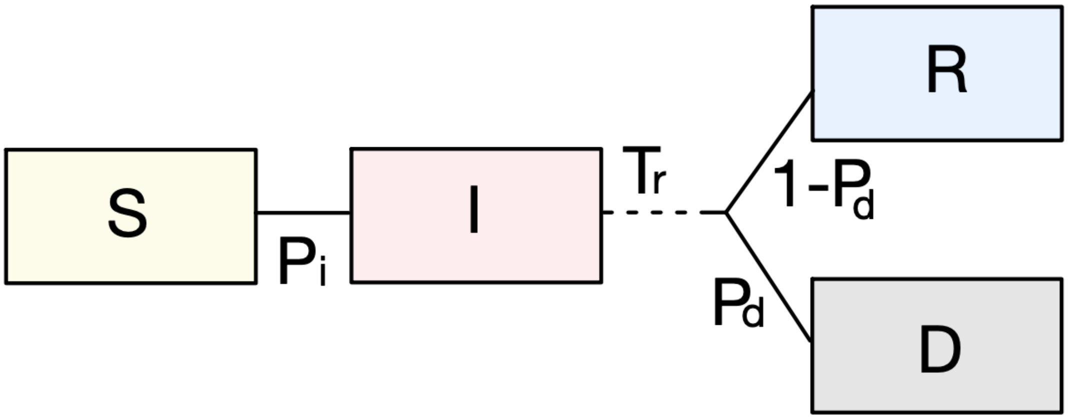


**S2 Fig.** Transition rates of our SIR model. Susceptible individuals can become infected with probability $P_i$if they have an infected neighbour. Infected individuals remain infected for an exponential random time with mean $T_r$. At the end of this infectious period they can recover with probability $1 - P_d$ or die with probability $P_d$ .

medRxiv preprint doi: https://doi.org/10.1101/2021.03.11.21253348; this version posted May 25, 2021. The copyright holder for this preprint
(which was not certified by peer review) is the author/funder, who has granted medRxiv a license to display the preprint in perpetuity.
It is made available under a CC-BY 4.0 International license .

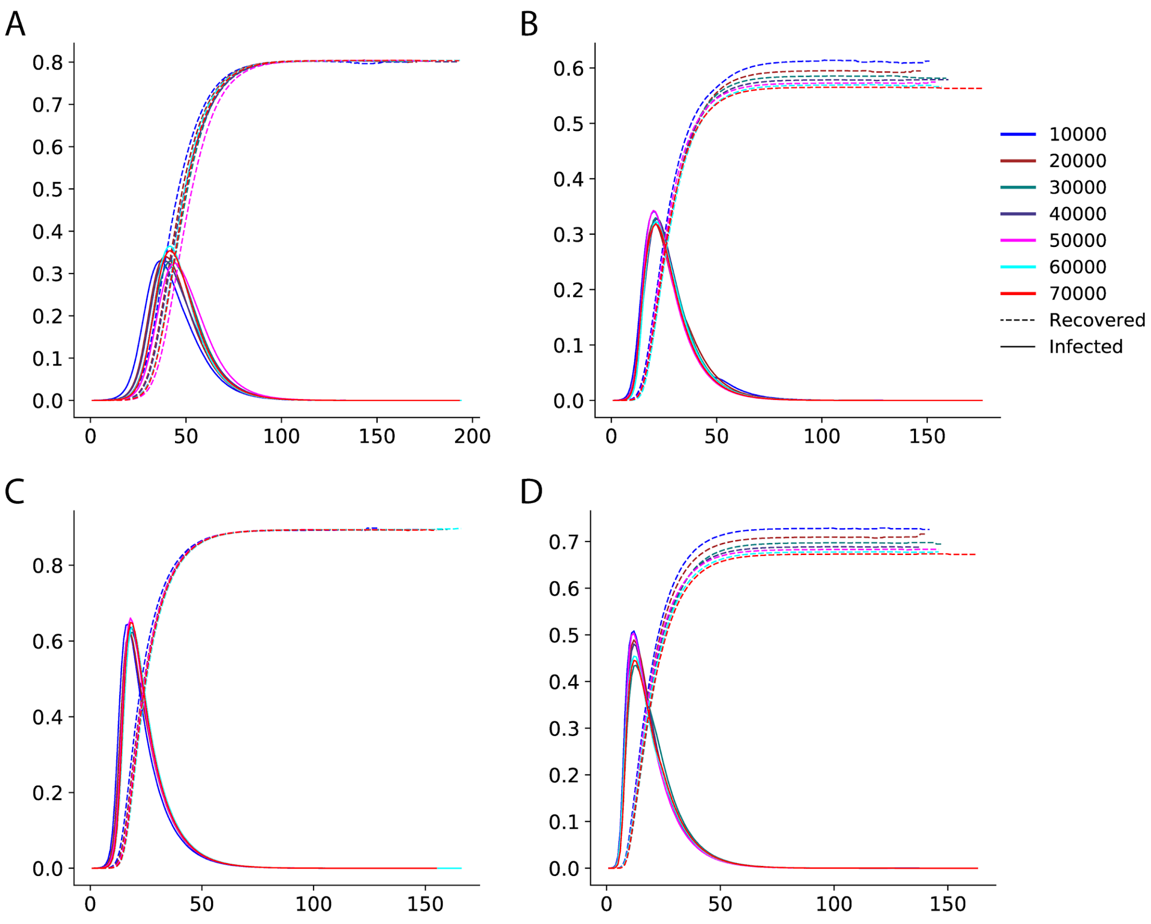


**S3 Fig.** Infected and recovered curves for population sizes 10 000, 20 000, 30 000, 40 000, 50 000, 60 000 and 70 000. The curves represent the proportion of individuals in each category as functions of time. Each curve corresponds to the mean over 30 repetitions. A, C: Erdős-Rényi graphs, B, D: Power-law degree, A, B: e = 5. C, D: e = 10. The rest of the parameters are $p_i$= 0.5 and $\lambda$ =3.

medRxiv preprint doi: https://doi.org/10.1101/2021.03.11.21253348; this version posted May 25, 2021. The copyright holder for this preprint (which was not certified by peer review) is the author/funder, who has granted medRxiv a license to display the preprint in perpetuity.
It is made available under a CC-BY 4.0 International license .

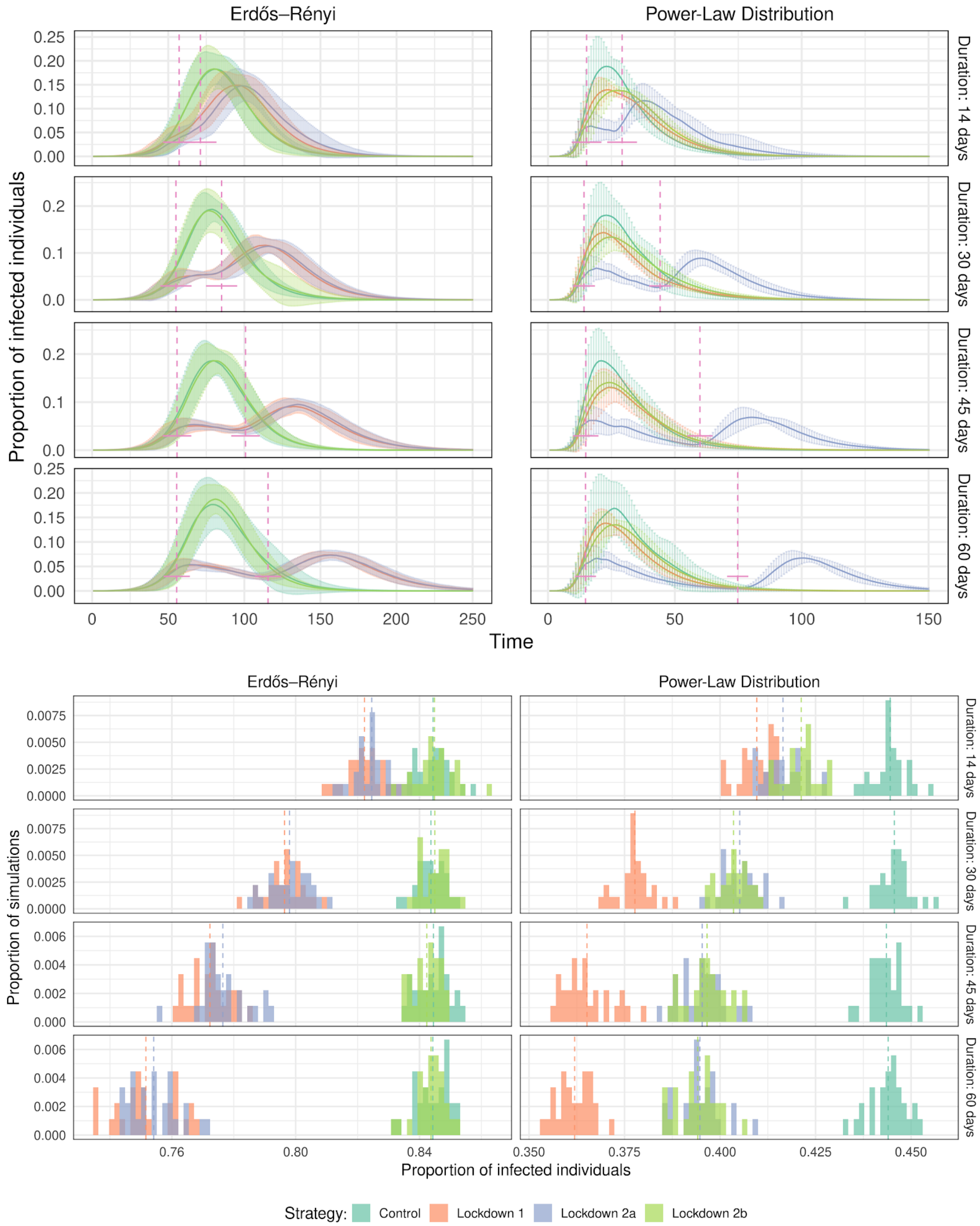


**S4 Fig.** Effect of the duration of the lockdown for different strategies. Top panels: proportion of infected individuals through time. Bottom panels: distribution of the total number of infected individuals for 30 different simulations. The lockdown starts when the cumulative number of infected individuals is 10%. In the top panels, the dashed lines show the beginning and the end of the lockdowns (and its standard deviation indicated by a horizontal line). In the bottom panel dashed lines correspond to the average proportion of infected individuals for each condition.

medRxiv preprint doi: https://doi.org/10.1101/2021.03.11.21253348; this version posted May 25, 2021. The copyright holder for this preprint (which was not certified by peer review) is the author/funder, who has granted medRxiv a license to display the preprint in perpetuity.
It is made available under a CC-BY 4.0 International license .

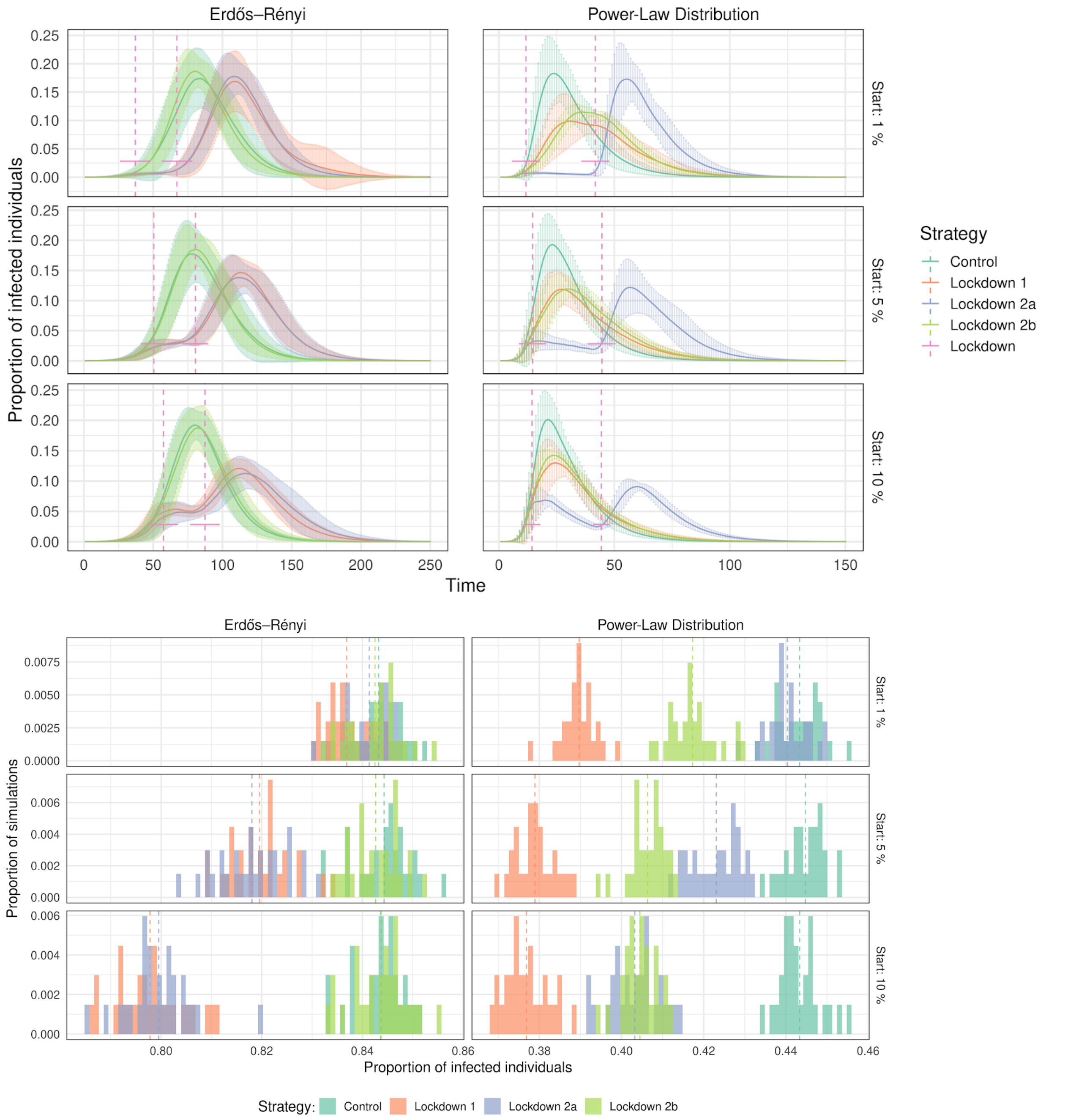


**S5 Fig.** Effect of the starting time for the lockdown. The duration is fixed as 30 days, and we vary the cumulative proportion of infected individuals at the start of the lockdown tL. Top panels: proportion of infected individuals through time. Bottom panels: distribution of the total number of infected individuals for 30 different simulations. The dashed lines have the same meaning as in S3 Fig. Observe that for tL=1%, lockdowns have no substantial effect on the maximum of the infection curve in the Erdős-Rényi case, and the same holds for lockdown strategy 2a in the power-law degree case. A lockdown started later (at tL=5% or 10%) is better with this respect.

medRxiv preprint doi: https://doi.org/10.1101/2021.03.11.21253348; this version posted May 25, 2021. The copyright holder for this preprint
(which was not certified by peer review) is the author/funder, who has granted medRxiv a license to display the preprint in perpetuity.
It is made available under a CC-BY 4.0 International license .

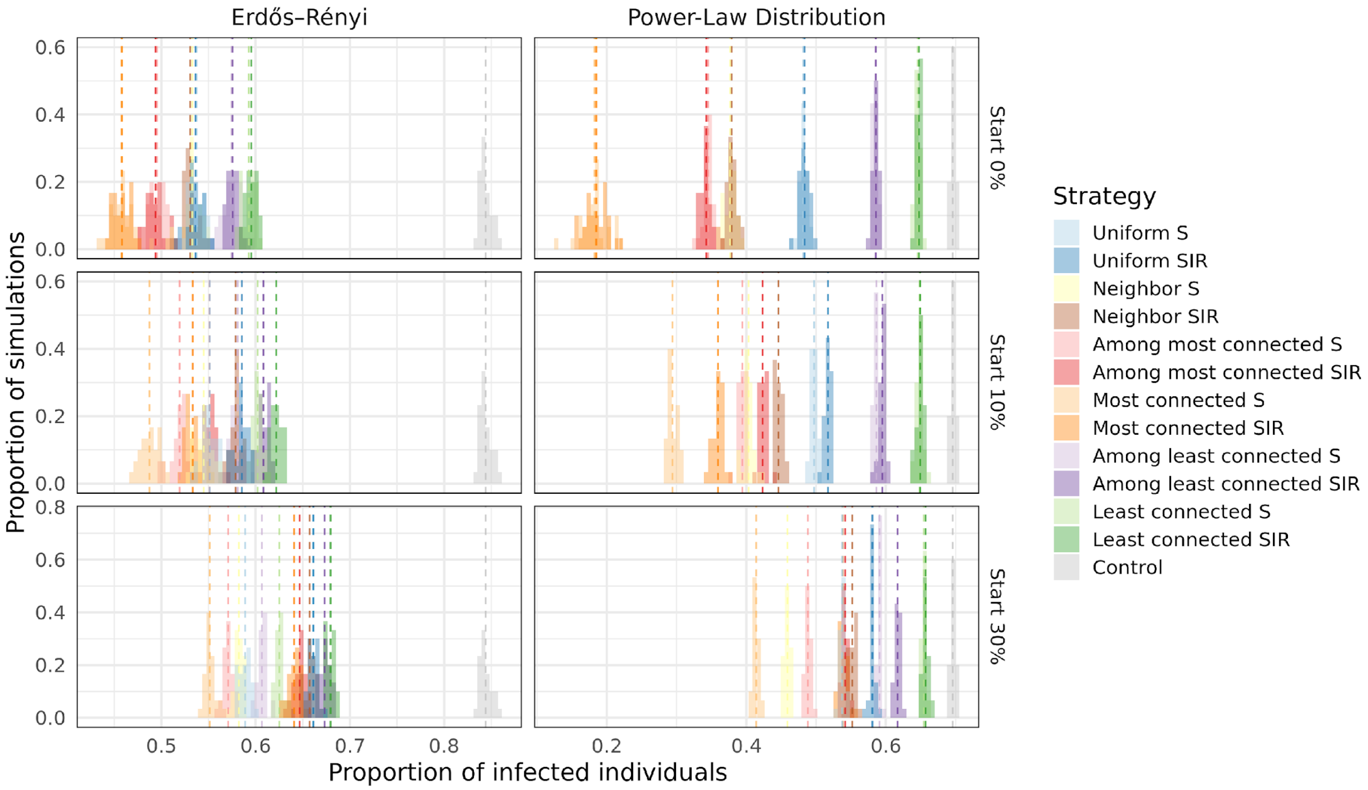


**S6 Fig.** Proportion of infected and dead individuals for all the vaccination strategies. The plot shows the proportion of infected at the end of the infection for 30 repetitions. The number of doses of the vaccine represents 25% of the population size (N = 20000). Different starting times are shown in the different panels (when 0, 10 and 30% of the individuals have been infected). On the top right panel, when vaccinating the most connected, the epidemic always died out quickly, before infecting at least 50 individuals, which is the minimum required to be considered a successful simulation (see Methods).

medRxiv preprint doi: https://doi.org/10.1101/2021.03.11.21253348; this version posted May 25, 2021. The copyright holder for this preprint
(which was not certified by peer review) is the author/funder, who has granted medRxiv a license to display the preprint in perpetuity.
It is made available under a CC-BY 4.0 International license .

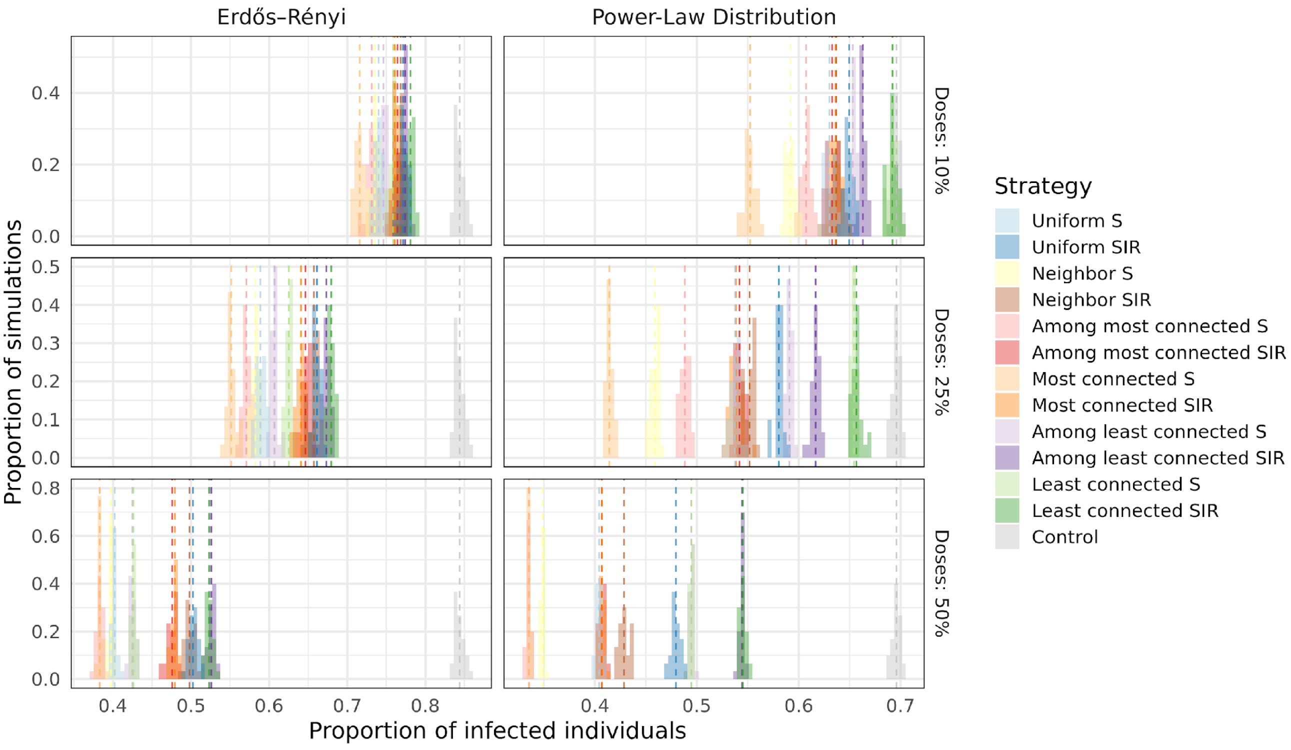


**S7 Fig.** Effect of the number of doses of the vaccine. Plots show the total number of infected individuals in 30 simulations for the Erdős-Rényi (right) and power-law (left) graphs. From top to bottom we increase the number of individuals that we can vaccinate (10, 25, 50%). The time of vaccination is when the cumulative number of infected reaches 30% of the populations ($t_V$= 30%).

medRxiv preprint doi: https://doi.org/10.1101/2021.03.11.21253348; this version posted May 25, 2021. The copyright holder for this preprint (which was not certified by peer review) is the author/funder, who has granted medRxiv a license to display the preprint in perpetuity.
It is made available under a CC-BY 4.0 International license .

## Supplementary Materials

### S1 Movie.

Propagation of the epidemics in an Erdős-Rényi graph. Vertices are colored depending on the status of the individual they represent. Blue: susceptible, red: infected, green: recovered, black: dead. The edge connecting $i$ and $j$ is colored in red when individual $i$ infects individual $j$. The numbers that appear at the end are the number of individuals that were infected by each individual.

### S2 Movie.

Propagation of the epidemics in a power-law degree distribution graph. Vertices are colored depending on the status of the individual they represent. Blue: susceptible, red: infected, green: recovered, black: dead. The edge connecting $i$ and $j$ is colored in red when individual $i$ infects individual $j$. The numbers that appear at the end are the number of individuals that were infected by each individual.
